# Alexithymia and Hypertension: Does Personality Matter? A Systematic Review and Meta-analysis

**DOI:** 10.1007/s11886-023-01894-7

**Published:** 2023-05-22

**Authors:** Marialaura Di Tella, Agata Benfante, Lorenzo Airale, Lorys Castelli, Alberto Milan

**Affiliations:** 1 Department of Psychology, University of Turin, Via Verdi 10, 10124 Turin, Italy; 2 Internal Medicine and Hypertension Division, Department of Medical Sciences, AOU Città Della Salute E Della Scienza Di Torino, University of Turin, Via Genova 3, 10126 Turin, Italy

**Keywords:** Alexithymia, Hypertension, Psychological factors, Systematic review, Meta-analysis

## Abstract

**Purpose of review:**

Personality characteristics, such as alexithymia, may lead to alterations in the autonomic nervous system functionality, predisposing individuals to an increased risk of hypertension (HTN). The present meta-analysis aimed to quantify the presence of alexithymia in people with HTN and to assess for potential sources of heterogeneity between studies. PubMed, PsycINFO and Scopus databases were systematically searched, using the following strings: (“alexithymia” OR “alexithymic”) AND (“hypertension” OR “hypertensive”). Data were meta-analyzed with random-effects models.

**Recent findings:**

A total of 13 studies met the inclusion criteria. The prevalence of alexithymia in people with and without HTN were obtained from 5 studies (26.3% vs 15.0%; pooling of odd ratios, 3.15 [95% CI, 1.14;8.74]), whereas the mean level of alexithymia between people with and without HTN was obtained from 7 studies Hedges g, 1.39 [95% CI, -0.39;3.16]). There was a significant association between alexithymia prevalence and year of article publication (ĝ = -0.04; 95% CI, -0.07;-0.01), whereas no significant relationship was detected between the former and both sex and age.

**Summary:**

Findings revealed a greater prevalence of alexithymia in people with HTN than in participants without HTN. These findings suggest that alexithymia may contribute to both the onset and persistence of HTN symptomatology. However, future research is needed to clarify this association.

**Supplementary Information:**

The online version contains supplementary material available at 10.1007/s11886-023-01894-7.

## Introduction

Hypertension (HTN) is a global public health concern, with 1.13 billion people currently diagnosed with stable high (or raised) blood pressure worldwide [1]. It represents a major cause of premature death and is a known factor for increased risk of heart, brain, kidney, and other diseases, especially for those cases in which adequate control of blood pressure is not implemented [e.g., 2–4].

HTN has a multifactorial etiology, with a number of genetic and behavioral factors that have been associated with increased blood pressure, such as obesity, insulin resistance, high alcohol intake, high salt intake, aging, sedentary lifestyle, and low potassium/calcium intake [e.g., 5]. Growing evidence is showing that psychological factors may also play an important role in the onset and maintenance of HTN. In particular, anger, anxiety/depressive symptoms, acute stress, and specific personality characteristics (e.g., type D and type A personality) have been frequently reported in people with HTN [6–9]. Among those psychological dimensions, personality traits deserve special attention for two main reasons. On the one hand, personality patterns can enhance behavioral risk factors for HTN, such as smoking, alcohol intake, obesity, and negative lifestyle. On the other hand, the presence of certain personality characteristics, together with other psychological symptoms, may lead to alterations in the autonomic nervous system functionality, predisposing individuals to an increased risk of high blood pressure [e.g., 10].

Alexithymia is a multidimensional personality construct, characterized by different cognitive and emotional features. The term alexithymia (literally, ‘no words for feelings’) was first coined by Sifneos in 1972 to describe individuals who lack the ability to communicate their feelings or have limited imagination [11, 12]. The main aspects of alexithymia are a difficulty in identifying and describing subjective feelings, a difficulty distinguishing between feelings and the bodily sensations of emotional arousal, restricted imagination processes, as evidenced by the lack of imagination, and a stimulus-bound, externally oriented cognitive style [13].

The ability to correctly identify one’s own emotions has been suggested to be essential for adequate emotion regulation processes, with deficits in this area that may occur as a chronic increase in autonomic arousal [14]. Previous psychophysiological studies showed, in fact, that individuals with high levels of alexithymia reported a raised resting sympathetic tone or a greater heart rate or blood pressure reactivity to stress-inducing stimuli [e.g., 15, 16]. Furthermore, positive correlations between alexithymia and norepinephrine/cortisol ratio have been detected in male individuals [17]. Excessive activation of the sympathetic nervous system may expose individuals to an increased risk of HTN in the long term.

Although previous studies have been conducted trying to address the levels of alexithymia in people with HTN [e.g., 18••, 19, 20••], to the best of our knowledge, no systematic review and meta-analysis has yet been carried out to synthesize those results. Therefore, the present systematic review and meta-analysis was conducted to address the following objectives: 1) To quantify the presence of alexithymia in people with HTN; 2) To clarify if the prevalence and mean level of alexithymia are higher in people with HTN than in individuals without HTN; 3) To examine the possible influence of specific factors (i.e., sex, age, and year of article publication) on alexithymia.

## Methods

A systematic review and meta-analysis was performed in line with the guidelines of the Preferred Reporting Items for Systematic Reviews and Meta-analysis Protocols (PRISMA) [21, 22], in order to synthesize the results of the literature on alexithymia in individuals with HTN.

### Search Sethod

**Fig. 1:**
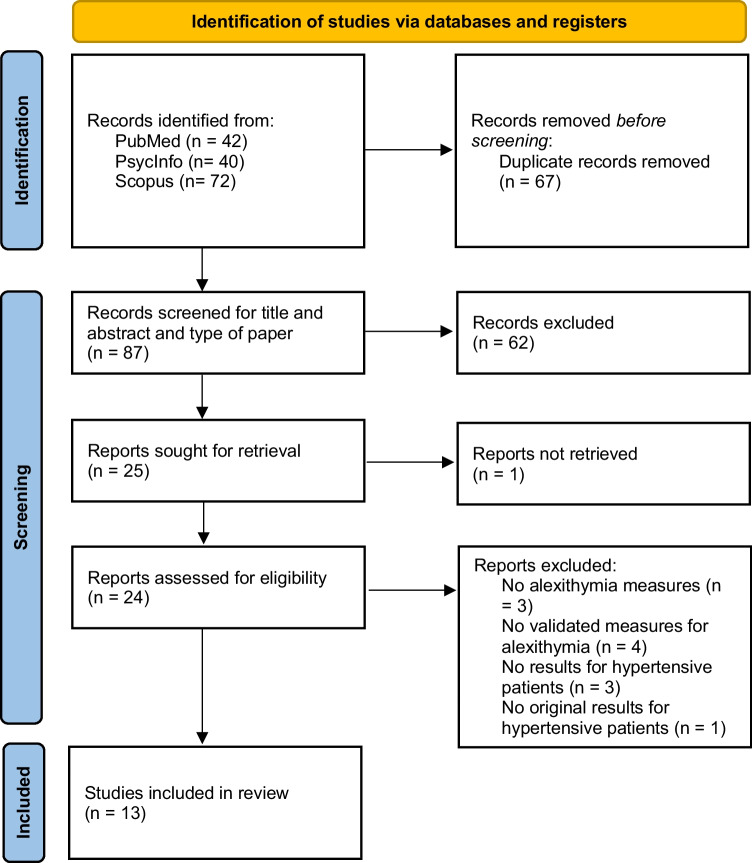
PRISMA 2020 flow diagram for new systematic reviews which included searches of databases and registers only [21]

A literature search was conducted in the first two weeks of June 2022, in the following bibliographic databases: PubMed, PsycINFO and Scopus. The following string with Boolean operators was used to search databases: (“alexithymia” OR “alexithymic”) AND (“hypertension” OR “hypertensive”). The final search was conducted on June 23, 2022. In this way, 154 records were identified. These articles were published between 1980 and 2022 (see Fig. 1 for the flow diagram of article selection). No additional article has been found using cross references.

### Eligibility Criteria

Inclusion criteria were further defined using the PICOS scheme (Participants (P), Interventions (I), Comparisons (C), Outcomes (O), Study Designs (S) [23]. Studies reporting the following data were included: adults with HTN (P), who received any type of treatment provided for this condition (I), and having or not individuals without HTN as comparison groups (C). Moreover, the presence of alexithymia and its implication was the outcome considered (O), and cross-sectional and longitudinal study design were both taken into consideration for this review (S).

Finally, peer-reviewed research papers (such as original articles, brief report, letters to editors), published in English and Italian, were included in this review.

Conversely, the following exclusion criteria were established:

- Papers that used ad hoc constructed surveys or qualitative methods (not validated) to assess alexithymia.
- Studies that included blood pressure assessment, but without considering clinically significant levels (i.e., hypertension).
- Studies that included patients with other cardiovascular diseases or metabolic syndrome without focusing on HTN.
- Articles such as case reports, study protocols and meeting abstracts that did not contain complete information.
- Papers not peer reviewed (i.e., gray literature) or under review at the time the search was conducted.

### Studies Screening and Selection

Two authors (AB and MDT) performed the studies selection. The two authors first screened the articles according to their titles and abstracts, then they read the full text of the articles selected, for further skimming.

Literature search has been conducted again by another author (LA), according to the steps described above, to ensure that the results of the search are replicable.

The authors discussed and reached agreement in the case of disagreements on the inclusion or exclusion of articles.

### Data Extraction

The information extracted from the studies was determined by all authors. AB and MDT collected the data independently and then interactively discussed the results. Authors, year of article publication, participants (sex and mean age), alexithymia measures, mean score and/or prevalence of alexithymia, other variables and measures used (i.e., sociodemographic, clinical, and psychological data), and the main results of the studies, were extracted from selected studies.

### Quality Assessment

The quality assessment of the included studies was performed by two independent assessors (MDT and AB) using the Joanna Briggs Institute (JBI) [24] critical appraisal tool for cross-sectional studies (studies with only one target group), case control studies (studies with at least one control group), and prevalence studies (studies reporting prevalence data only). Those checklists include 8, 10, and 9 questions, respectively. Possible answers for each question are yes, no, unclear, and not applicable. Scores for overall quality range from 0 to 8 for cross-sectional studies, from 0 to 10 for case-controlled studies, and from 0 to 9 for prevalence studies. Disagreements on the scoring to be assigned were discussed by all authors until agreement was reached.

### Data Analysis

Statistical analysis was conducted on the summary statistics described in the selected articles. Pooled prevalences were analyzed through logit transformation and effect was pooled through random intercept logistic regression model.

Prevalences comparison between groups were analyzed through logit transformation and effect was pooled through Mantel–Haenszel method. Moreover, standardized mean differences (effect sizes) were calculated for overall alexithymia using Hedges g. This represents the difference between the means of the HTN and no HTN groups, divided by the pooled SD and weighted for sample size. Additional supplementary analyses were also conducted to examine performance on the 3 types of alexithymia assessment used by the included studies: the 20-item and 26-item versions of the Toronto Alexithymia Scale and the Toronto Structured Interview for Alexithymia.

The proportion of variability explained by true heterogeneity (i.e., between-studies variability) was estimated by calculating the I^2^ for each analysis. All the pooled effects were computed by random-effect models because of high heterogeneity among the studies and Hartung-Knapp adjustment was used.

Funnel plot was used to detect and examine publication and small study biases. The funnel plot asymmetry was statistically checked using Egger’s test [25], when at least 10 studies were included [26]. Accordingly, asymmetry of the funnel plot and/or statistical significance of Egger’s test (*p* < 0.05) were suggestive of publication bias or small study effects.

Exploratory meta-regression analyses were finally conducted to examine the associations between alexithymia and a set of variables that were available in all the selected studies, and to detect potential sources of heterogeneity. The following variables from the HTN group were identified a priori and examined in univariate analyses: age, sex (proportion of each sample that were female), and year of article publication. Alexithymia was expressed in the analyses as pooled prevalence, because of the variety in the use of measures by the selected studies (e.g., some of these instruments were used by one study only).

All meta-regression analyses were performed using a random effects model. Meta-regression coefficients and corresponding *p*-values have been reported.

All statistical analyses were performed with R software—version 4.1.1 [27] with “meta” [28] and “metafor”—version 3.4 [29] packages.

## Results

### Study Celection

Based on our retrieval strategy, 154 records were found in databases searching. After duplicates removal, AB and MDT screened 87 records for title and abstract. Only one article was not included because the full-text version was not accessible, while the others were excluded because they did not meet the eligibility criteria (n = 62).

AB and MDT reviewed 24 full-text documents. Of those, 11 articles were excluded for the following reasons: alexithymia was not assessed in the study (n = 3), alexithymia was assessed with not validated instruments or only by clinical consultation (n = 4), participants did not have hypertension (n = 3), data on patients with hypertension were taken from another study (n = 1). Citations for the excluded articles are listed in supplementary material (see File, Supplementary Material 1). Finally, 13 studies were included in this review.

### Study Characteristics

A summary of the main characteristics and results of the 13 studies included in the present systematic review is provided in Table 1.

**Table 1:** Summary of the main characteristics and findings of the included articles (n = 13)

Authors (years)	Participants (M/F, mean age)	Alexithymia Measures	Other Measures	Main results of alexithymia in HTN participants	Risk bias
Casagrande et al. (2019b) [18••]	810 with HTN (434 F; mean age: 60.1, SD: 11.2) 431 without HTN (273 F; mean age: 51.5, SD: 10.2)	TAS-20	*Socio-demographic and clinical data*: age, gender, marital status, years of education, smoking, alcohol consumption, BMI, SBP, DBP, mean arterial pressure, heart rate, and clinical information *Adherence to drug*: ad hoc yes/no questionnaire	Alexithymia prevalence in HTN participants: 16.4% (133). Alexithymia mean (SD) score in HTN participants: 49.01 (12.10) HTN participants reported higher levels of alexithymia compared to individuals without HTN (normotensive individuals), even after controlling for age, education, and BMI In addition, treated HTN reported higher alexithymia scores compared to both untreated participants with and without HTN	Low
Consoli et al. (2010) [19]	98 with HTN: 73 with essential HTN, 25 with secondary HTN (62 F, mean age essential HTN: 53.4, SD essential HTN: 12.7; mean age secondary HTN: 52.2, SD secondary HTN: 15.7)	TAS-20	*Socio-demographic and clinical data*: age, gender, education, disease duration, smoking, BMI, SBP, DBP, and clinical information *Emotion awareness*: LEAS *Coping*: WCC	Alexithymia prevalence in essential HTN vs. secondary HTN patients: 25% (18) vs. 20% (5) Alexithymia mean (SD) score in essential HTN vs. secondary HTN patients: 54.2 (11.6) vs. 48.4 (12.1) No statistically significant differences between essential HTN and secondary HTN patients either in alexithymia scores or in alexithymia percentages	Low
Di Trani et al. (2018) [30]	15 with HTN (8 F; mean age: 48.5, SD: 10.7) 20 without HTN (13 F; mean age: 25, SD: 3.8)	TAS-20 TSIA	*Socio-demographic data*: age and gender *Linguistic measures*: WRAD, Italian reflection dictionary, disfluency dictionary, and somatic sense	Alexithymia mean (SD) score in HTN participants: TAS: 40.23 (9.92); TSIA: 28.20 (6.03) HTN participants did not report higher levels of alexithymia on the TAS-20 compared to individuals without HTN, whereas they yielded higher scores on the TSIA than individuals without HTN	High
Gage and Egan (1984) [36]	66 with HTN (34 F; mean age and SD: N.A.) No control group	MMPI	*Socio-demographic data*: age, gender, DBP ≥ 95 mm Hg	Alexithymia prevalence in HTN participants: 47% (31) In alexithymic HTN participants the severity of HTN was greater than in non-alexithymic HTN participants	High
Grabe et al. (2010) [39]	503 with HTN (247 F; mean age: 51.0, SD: 0.4) 675 without HTN (417 F; mean age: 42.7, SD: 0.4)	TAS-20	*Socio-demographic and clinical data*: age, gender, education, marital status, smoking, physical activity, BMI, SBP, DBP, and clinical information *Mental distress*: CID-S	Alexithymia prevalence in HTN participants: 23.9% (120) Alexithymia mean (SD) score in HTN participants: 42.6 (0.4) HTN participants yielded higher alexithymia scores and were found to be more alexithymic (score ≥ 49) compared to individuals without HTN	Low
Hänninen et al. (2011) [33]	744 with HTN: 221 with white-coat HTN, 118 with masked HTN, 405 with sustained HTN (white-coat HTN: 107 F; mean age: 56.2, SD: 8.1; masked HTN: 44 F; mean age: 56.9, SD 8.1; sustained HTN: 187 F; mean age: 58.6, SD: 8.5) 715 without HTN (426 F; mean age: 53.9, SD: 7.2)	TAS-20	*Socio-demographic and clinical data*: age, gender, education, marital status, smoking, physical activity, alcohol consumption, BMI, office SBP and DBP, home SBP and DBP, and clinical information *Psychopathological symptoms*: Whiteley-7, BDI, GHQ-12	Patients with sustained HTN reported higher alexithymia scores compared to patients with white-coat HTN	Low
Jula et al. (1999) [34]	237 with HTN (99 F; mean age: 45.7 for M, 46.4 for F; SD: 5.1 for M, 4.7 for F) 146 without HTN (78 F; mean age: 44 for M, 44.2 for F; SD: 5.3 for M, 5.4 for F)	TAS-26	*Socio-demographic and clinical data*: age, gender, socioeconomic class, BMI, alcohol intake, smoking, SBP, DBP, clinical information *Psychopathological symptom:* BSI-37, STAXI	Alexithymia prevalence in HTN participants: 57% (79) men; 46% (46) women Alexithymia mean (SD) score in HTN participants: 45.7 (5.1) men; 46.4 (4.7) women HTN participants have significantly higher TAS mean scores and a higher prevalence of alexithymia than individuals without HTN. HTN women had more somatization symptoms than women HC	Low
Muneta et al. (1997) [38]	101 with HTN: 48 whit white-coat HTN, 53 with sustained HTN (white-coat HTN: 34 F, mean age: 59.2, SD: 9.5; 14 M, mean age: 60.1, SD: 11.5; sustained HTN: 32 F, mean age: 58.6, SD: 9.7; 21 M, mean age: 59.5, SD: 13)	MMPI	*Socio-demographic and clinical data:* age, gender, clinic SBP and DBP, home SBP and DBP *Psychological characteristics and symptoms:* SRQ-D, Type A Behavior Pattern Questionnaire, GHQ, ECL	Alexithymia prevalence: in white-coat HTN participants, 14.3% (2) men; 26.5% (9) women; in sustained HTN participants, 4.8% (1) men, 31.3% (10) women Alexithymia mean (SD) score: in white-coat HTN participants, 12.9 (4.6) men; 15.2 (1.9) women; in sustained HTN participants, 13.7 (1.8) men, 15.8 (2.3) women White-coat HTN participants did not differ from sustained HTN in alexithymia scores	Medium
Niiranen et al. (2006) [35]	593 with HTN: 371 with sustained HTN, 222 with isolated clinical HTN (sustained HTN: 169 F, mean age: 57.9, SD: 8.6; isolated clinical HTN: 108 F, mean age: 55.8, SD: 8.3) 847 without HTN (370 F; mean age: 53.5, SD: 7.6)	TAS-20	*Socio-demographic and clinical data*: age, gender, education, smoking, BMI, clinic blood pressure (SBP, DBP), clinical information *Psychopathological symptoms*: Whiteley-7, BDI, GHQ-12	Alexithymia mean (SD) score: in sustained HTN participants, 48.2 (10.9); in isolated clinical HTN participants, 45.2 (10.8) Sustained HTN participants had higher scores of alexithymia and were more alexithymic than those with isolated clinical HTN and without HTN	Low
Paulson (1985) [37]	53 with HTN (4 F; mean age: 56.5, SD: 6.5) No control group	BIQ MMPI Schalling-Sifneos Scale	*Socio-demographic and clinical data:* age, gender, marital status, education, clinical information	Alexithymia prevalence in HTN participants: 41% (22) (BIQ) No differences were found between alexithymic and non-alexithymic participants for socio-demographic and clinical data. No correlation was found between BIQ and other scales used	Medium
Piotrowska-Półrolnik et al. (2019) [20••]	39 with HTN (23 F, mean age: 42.9, SD: 13.5) 37 without HTN (26 F, mean age: 36.7, SD: 11.5)	TAS-20	*Socio-demographic and clinical data*: age, gender, education, marital status, BMI, clinical information, 24 h-ABPM	Alexithymia prevalence in HTN participants: 17.9% (7). Alexithymia mean (SD) score in HTN participants: 48.33 (N.A.) Alexithymia mean level was higher in HTN participants than in individuals without HTN. There were statistically significant positive correlations between higher alexithymia levels and higher 24-h SBP values	Medium
Rafanelli et al. (2012) [31]	125 with HTN (52 F, mean age: 66.4, SD: 11) No control group	DCPR	*Socio-demographic and clinical data*: age, gender, education, marital status, working status, clinical information, smoking, family risk, blood pressure ranging *Psychological characteristics and symptoms*: SCID, PSI, SQ, PWB scale	Alexithymia prevalence in HTN participants: 36% (45). The alexithymia cluster consisted of 55 patients (68.7%). HTN participants with moderate to severe levels of HTN have been more represented in the anxiety-depression and alexithymia clusters	Low
Todarello et al. (1995) [32]	114 with HTN (64 F, mean age: 52.5, SD: 11) 113 Psychiatric patients 130 without HTN (81 F, mean age: 40.8, SD: 16.7)	TAS-20	*Socio-demographic and clinical data*: age, gender, education, clinical information, SBP, DBP	Alexithymia prevalence in HTN participants: 55.3% (63). Alexithymia mean (SD) score in HTN participants: 61.99 (13.93) HTN participants had significantly higher levels of alexithymia than psychiatric patients, which had higher scores than individuals without HTN. A significantly higher rate of alexithymia was found in the HTN group than other two groups	Medium

*HTN *Hypertension, *TAS *Toronto Alexithymia Scale, *BMI *Body Mass Index, *LEAS *Levels of Emotional Awareness Scale, *WCC *Ways of Coping Checklist, *TSIA *Toronto Structured Interview for Alexithymia, *WRAD *Weighted Referential Activity Dictionary, *NA *Not Available, *MMPI *Minnesota Multiphasic Personality Inventory, *CID-S *Composite International Diagnostic-Screener, *BDI* Beck Depression Inventory, *GHQ* General Health Questionnaire, *SBP* Systolic Blood Pressure, *DBP* Diastolic Blood Pressure, *BSI-37* Brief Symptoms Inventory, *STAXI* 31 item Spielberger State-Trait Anger Expression Inventory, *SRQ-D* Self Rating Questionnaire for Depression, *ECL* Egogram Check List, *BIQ* Beth Israel Hospital Questionnaire, *ABPM* Ambulatory Blood Pressure Monitoring, *DCPR* Diagnostic Criteria for Psychosomatic Research, *SCID* Structured Clinical Interview for DSM-IV, *PSI* Psychosocial Index, *SQ* Symptom Questionnaire, *PWB* Psychological Well-Being Scale

The included studies were cross-sectional studies (6), case control studies (4), and prevalence studies (3). The publication year ranges from 1984 to 2019.

Regarding the study area, 4 studies were conducted in Italy [18••, 30–32], 3 studies in Finland [33–35], 2 in the United State of America [36, 37], 1 in Japan [38], 1 in Germany [39], 1 in France [19], and 1 in Poland [20••].

Taking all studies together, the participants with HTN amount to 3,498, with sample sizes ranging from 15 [30] to 810 [18••]. Of those participants, 1,708 were female and the mean age was between 42.86 [20••] and 66.4 [31] years.

Regarding individuals without HTN, the participants amount to 3,000, with sample sizes ranging from 20 [30] to 847 [35]. Of those individuals 1,603 were female and the mean age was between 25 [30] and 53.9 [33] years.

Many of the studies evaluated patients’ clinical data (i.e., blood pressure value, smoking). Conversely, only 5 studies assessed psychological characteristics and symptoms (i.e., anxiety, depression, personality traits) with various questionnaires. The presence of alexithymia was assessed with the following different instruments: observer-rated measures, such as the Diagnostic Criteria for Psychosomatic Research (DCPR) [31] and Toronto Structured Interview for Alexithymia (TSIA) [30]; and self-report measures, such as the Toronto Alexithymia Scale (TAS) [18••, 19, 20••, 30, 32–35, 39], Beth Israel Hospital Questionnaire (BIQ) [37], Shalling-Sifneos Scale [37], and Minnesota Multiphasic Personality Inventory (MMPI) [36–38].

### Prevalence of Alexithymia in HTN

With regard to the alexithymia prevalence in HTN, data were obtained from 10 studies [18••, 19, 20••, 31, 32, 34, 37–39]. The other 3 studies were not included because information about prevalence was not available.

**Fig. 2:**
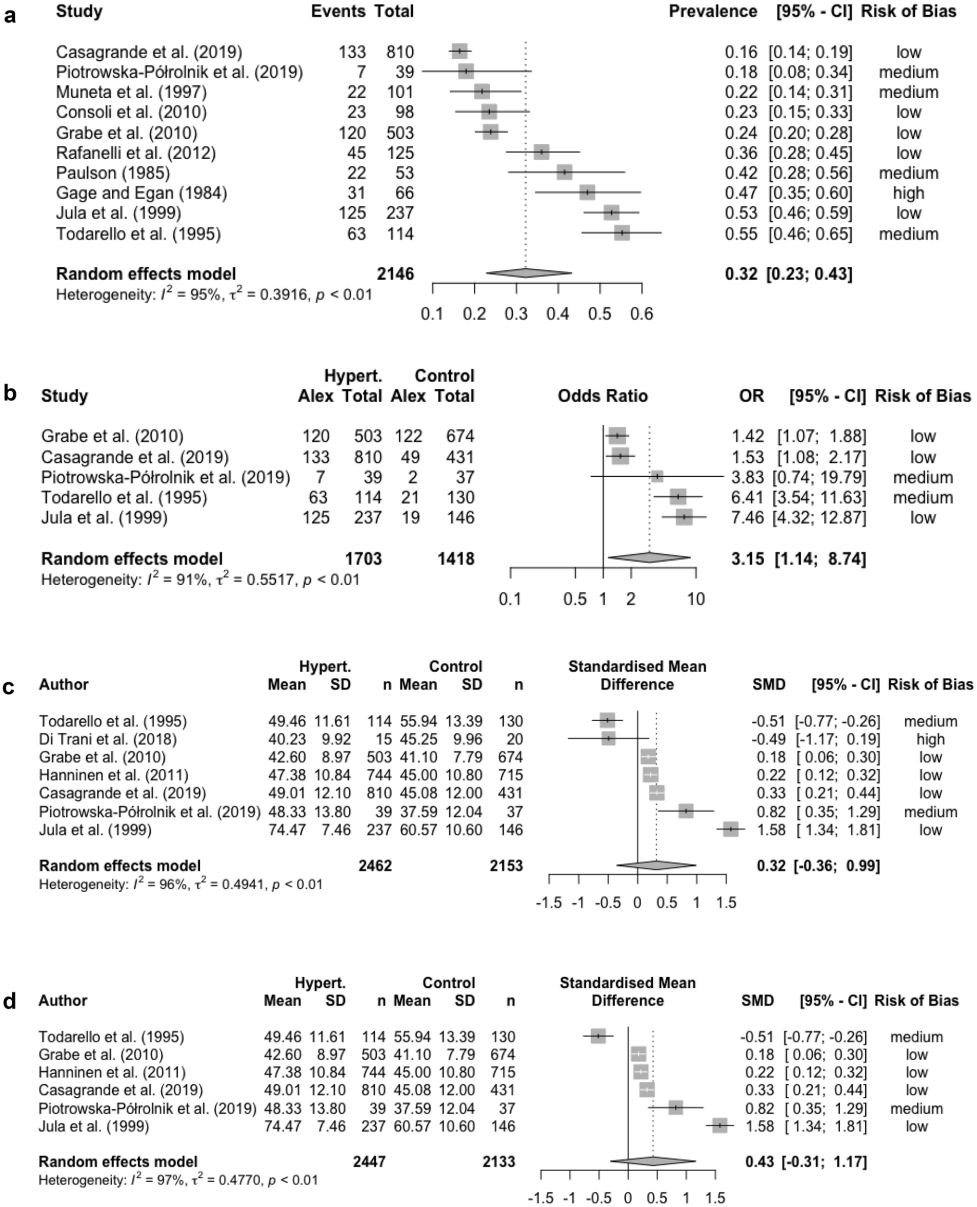
**(a)** Forest plot of the pooled prevalence of alexithymia in people with HTN. **(b)** Forest plot of the pooling of odd ratios (ORs) of alexithymia prevalence in people with vs. without HTN. **(c)** Forest plot of the standardized mean difference (SDM) of alexithymia in people with vs. without HTN; **(d)** Forest plot of the SDM of alexithymia in people with vs. without HTN (sensitivity analysis)

The prevalence of alexithymia in HTN was based on the percentage of individuals who scored above the cut-off values for the different instruments that studies employed. The reported overall prevalence of alexithymia in HTN ranged from 16.4% [18••] to 55.3% [32], and the pooled random effects prevalence was 32.2% (95% CI, 22.81;43.29). There was a high heterogeneity between studies as evidenced by I^2^ = 95.1%. The estimate of between-study variance (Tau-squared) was 0.39 (Fig. 2a).

In order to ascertain possible publication bias, a funnel plot was plotted by labeling the transformed log odds ratio (transformed log ORs) of the effect size to the x-axis and the standard error of the transformed log ORs to the y-axis. There was no significant publication or small study effect as evidenced by the symmetrical funnel plot and insignificant Egger’s test (3.61, 95% CI -3.17;10.4, *t* = 1.04, *p* = 0.33) (see Figure a, Supplementary Material 2).

### Comparison Between People with and without HTN on the Prevalence and Mean Level of Alexithymia

With regard to alexithymia prevalence, data were obtained from 5 studies [18••, 20••, 32, 34, 39] that compared the prevalence of alexithymia in people with and without HTN. Three other studies, which included control groups without HTN, were not inserted because the necessary data could not be obtained from the authors [30, 33, 35].

People with HTN were more likely to be alexithymic than participants without HTN (26.3% vs 15.0%; pooling of ORs, 3.15 [95% CI, 1.14;8.74]; Fig. 2b). High heterogeneity between studies was detected, as evidenced by I^2^ = 87.5%.

In order to assess publication bias, a funnel plot was plotted by labeling the log risk ratio of the effect size to the x-axis and the standard error of log risk ratio to the y-axis. Based on the few studies included, the funnel plot does not show significant publication or small study effect (see Figure b, Supplementary Material 2).

As far as the alexithymia mean level was concerned, data were obtained from 7 studies [18••, 20••, 30, 32–34, 39] that compared the standardized mean difference of alexithymia in people with and without HTN.

Alexithymia mean levels were not found to be significantly higher in HTN individuals than in participants without HTN (participants with vs. without HTN, alexithymia means: 50.3 vs. 47.3, respectively; Hedges g, 1.39 [95% CI, -0.39;3.16]; Table 2 and Fig. 2c). The high heterogeneity between studies was confirmed, as evidenced by I^2^ = 100%. A sensitivity analysis was also conducted excluding Di Trani et al.’s study [30], which was characterized by a high risk of bias (or a low quality) (participants with vs. without HTN, alexithymia mean: 51.9 vs. 47.6; Hedges g, 0.43, 95% CI, -0.31;1.17, I^2^ = 97%; Fig. 2d).

**Table 2:** Mean weighted effect sizes, sample sizes, and heterogeneity statistics

Test	No. of studies	No. (with HTN)	No. (without HTN)	Hedges g	95% CI	I^2^ (95% CI)
Alexithymia (overall)	7	2477	2095	1.39	(-0.39; 3.16)	99.5 (99.4; 99.6)
TAS-20	6*	2225	2007	1.47	(-1.16; 4.11)	99.7 (99.6; 99.7)
TAS-26	1	237	68	1.28	(0.99; 1.57)	–
TSIA	1**	15	20	1.01	(0.29; 1.72)	–

*HTN *Hypertensive, *CI *confidence interval, *TAS *Toronto Alexithymia Scale, *TSIA* Toronto Structured Interview for Alexithymia

^*^Niiranen et al. (2006) [35] were excluded from the analysis because of sample overlapping with Hänninen et al. (2011) [33]

^**^Di Trani et al. (2018) [30] employed both the TAS-20 and TSIA for the assessment of alexithymia

In order to assess publication bias, a funnel plot was plotted by labeling the standardized mean difference of the effect sizes to the x-axis and the standard error to the y-axis. Based on the few studies included, the funnel plot does not show significant publication or small study effect (see Figure c, Supplementary Material 2).

### Meta-Regression

To examine the possible influence of specific factors (i.e., year of article publication, sex and age) on the prevalence of alexithymia, three univariate meta-regressions were performed. Particularly, in addition to considering socio-demographic variables (age and sex), we have also taken into account a potential confounding factor, i.e. the year of article publication, given the advancements in the alexithymia measures that have been made over time.

With regard to the first univariate meta-regression, results showed a negative association between alexithymia prevalence and year of article publication (-0.04; 95% CI, -0.07;-0.01). Conversely, with regard to sex, results of the univariate meta-regression revealed no significant association between alexithymia prevalence and sex (-0.02; 95% CI, -0.05;0.01).

Similarly, results of the third univariate meta-regression showed no significant association between alexithymia prevalence and age (-0.01; 95% CI, -0.08;0.06).^1^

### Quality Assessment

The results of quality assessment for cross-sectional, case–control, and prevalence studies are presented in Supplementary Material 3 (File). The quality ratings ranged from 6 (75%) to 8 (100%) for cross-sectional studies (N = 6; M = 7.50, SD = 0.84; median = 8), from 5 (50%) to 9 (90%) for case–control studies (N = 4; M = 7.50, SD = 1.73; median = 8), and from 4 (44%) to 7 (78%) for prevalence studies (N = 3; M = 5.67, SD = 1.53; median = 6). Based on these results, 2 studies were classified as low quality, 4 studies as medium quality, and 7 studies as high quality.

## Discussion

The main aim of the present systematic review and meta-analysis was to summarize the available evidence on the association between alexithymia and HTN. Particularly, the following objectives were pursued: 1) to quantify the presence of alexithymia in people with HTN; 2) to compare the prevalence and mean level of alexithymia in people with and without HTN; 3) to examine the possible influence of specific factors (i.e., year of article publication, sex and age) on alexithymia.

With regard to the overall prevalence of alexithymia (based on the results of 10 studies), results showed a high prevalence of alexithymia (32%) in people with HTN.

Comparisons between people with and without HTN on alexithymia mean level revealed no statically significant differences between the two groups. Conversely, individuals with HTN were found to report a higher prevalence of alexithymia (26%) than participants without HTN (15%) (based on the results of 5 studies). These contrasting findings may rely on different explanations. First, some of the studies we included in the present meta-analysis could only be used for the estimation of difference in alexithymia mean level [30, 33], because additional data on alexithymia prevalence could not be retrieved from the authors. Second, high heterogeneity among the selected studies was detected. Particularly, most of those studies were characterized by a distinct methodology, with the inclusion of different sample sizes and the use of various instruments for the assessment of alexithymia. With regard to the latter aspect, different instruments for the assessment of alexithymia have been developed over time. However, while some of those instruments evaluate alexithymia among other personality characteristics (e.g., the MMPI) [40], other measures have been specifically conceived for the evaluation of alexithymia only (e.g., the TSIA [41] and the TAS [42]). These more recent instruments have been devised to overcome the limitations of pre-existing tools [43]. In fact, the MMPI, which was the most commonly employed instrument by our earlier studies, was developed with the general aim of assessing personality characteristics in medical and psychiatric syndromes. Subsequently, some items were used to create an alexithymia subscale score [40]. Conversely the TAS, which has been the most frequently used measure among the latest studies included in the present meta-analysis, provides investigators with a reliable, validated, and common metric for measuring the main facets of alexithymia specifically [44].

Although the role of alexithymia in HTN still needs to be clarified, the high prevalence of alexithymia we have found in people with HTN seems to suggest that this personality trait might characterize the HTN population. According to this assumption, the prevalence of alexithymia we detected in people with HTN is higher than that of both the participants without HTN included in the studies and the general population [45, 46].

In line with the present findings, high levels of alexithymia have also been identified in other clinical populations, in which patients appear to be characterized by great difficulties in identifying and describing their own feelings. For instance, a high prevalence of alexithymia was reported in people suffering from chronic pain, inflammatory bowel disease, diabetes and infectious diseases [47, 48, 49•, 50–52].

All those conditions are recognized as multidimensional phenomena in which biological, psychological and social factors dynamically interact with each other in determining the clinical expression of symptoms. Particularly, psychological aspects can contribute to both the onset and persistence of symptomatology. For instance, previous studies showed that uncontrolled hypertension, which is usually characterized by reduced adherence, unhealthy lifestyles and inadequate drug therapy [e.g., 53], is associated with specific psychological factors, such as coping strategies [54] and personality traits [55], including alexithymia [18••]. Similar results were obtained in people living with infectious diseases [52]. Sofia et al. [51] found, in fact, that alexithymia and poor emotion recognition were significantly associated with reduced adherence in people with HIV. Therefore, the presence of alexithymia might have an impact on both the maintenance of HTN, with possible low adherence to drug therapy, and the initial presentation of symptoms, with dysregulation of the autonomic nervous system that has been linked to various cardiovascular risk factors [10].

As far as the relationship between alexithymia prevalence and a series of possible influencing factors (i.e., year of article publication, sex and age) is concerned, three meta-regression analyses were performed to assess these associations. Results of the first meta-regression showed that the prevalence of alexithymia decreases as the year of article publication increases. A possible explanation for this finding may rely on the methodological rigor of studies, which has grown over time. Indeed, qualitative assessment revealed that the majority of recent studies obtained higher scores compared to older ones. For instance, as discussed above, newer studies included larger sample sizes of people with HTN (and without HTN), and employed more specific instruments, such as the TAS-20, compared to older studies.

A different pattern of results was detected for sex and age. Indeed, results of meta-regressions showed no significant association between both those variables and alexithymia prevalence. With regard to sex, although some previous studies conducted in the general population appear to show that men report higher alexithymic traits compared to women [e.g., 56], within the context of HTN those differences do not seem to apply. For instance, among the studies included in the present meta-analysis, which specifically addressed this issue, Jula et al. [34] and Muneta et al. [38] found small or no significant differences in alexithymic traits between men and women with HTN.

Similarly, alexithymia prevalence was not shown to vary with age in people with HTN. Those results could be explained taking into account the lack of heterogeneity in terms of age among patients with HTN. Indeed, the age of onset of HTN was generally over 55 years of age, with early-onset HTN that was defined as onset at age ≤ 55 years [57]. In line with this threshold, most of the included studies enrolled patients who had a mean age of 50 years or more, with other age groups (e.g., young adults) that were not represented. The available evidence seems to show that the prevalence of alexithymia increases steadily with advancing age in the general population [e.g., 58, 59]. For instance, the study of Mattila et al. [58] reported that 4.7% of youngest participants vs. 29.3% of oldest ones were alexithymic using the TAS-20. Similarly, Onor et al. [59] found a significant age effect on the TAS-20 total and subscale scores, with younger participants reporting lower scores than older ones. Those results might be due to the different cognitive and neurobiological-emotional changes associated with aging [e.g., 60]. Therefore, given the absence of young age groups in HTN, any differences in alexithymia prevalence might be difficult to detect.

The present systematic review and meta-analysis has also some limitations that should be acknowledged. First, the present systematic review determined the prevalence and mean level of alexithymia in the presence of high heterogeneity among individual studies, which limits the direct interpretation of pooled estimates. Second, other factors that might be associated with alexithymia were not taken into account due to the lack of data in the included studies. For this reason, we could only assess the relationship between the prevalence of alexithymia and a limited number of predictors (i.e., year of article publication, sex and age). Finally, the available studies that investigated the presence of alexithymia in people with HTN are still limited. Therefore, only a few studies have been included in the analyses, especially with regard to the comparison between people with and without HTN, restricting the generalizability of the present findings.

Despite those limitations, the current systematic review and meta-analysis represents, to the best of our knowledge, the first attempt to examine and quantify the relationship between alexithymia and HTN. The present findings show an increased prevalence of alexithymia in people with HTN compared to participants without HTN, although alexithymia mean level was not found to be significantly different between the two groups. Therefore, further research is needed to further clarify the association between alexithymia and HTN, overcoming the limitations of the available studies. Future longitudinal and intervention studies could provide new insight in the psychological and personality mechanisms that are involved in the genesis and maintenance of HTN. Particularly, a deeper understanding of the pathway through which alexithymia contributes to HTN may help physicians to plan better-tailored treatments. Indeed, if psychological aspects are recognised, a multidisciplinary approach to the treatment of HTN patients can be adopted.

For instance, in addition to the “treatment as usual”, psychological interventions might be implemented, focusing on improving the ability to identify emotions and on increasing adaptive emotional regulation processes in individuals with HTN. In this way, the maintenance of treatment adherence and the quality of life of these patients could be enhanced.

## Supplementary Information

Below is the link to the electronic supplementary material.

Supplementary file1 (DOCX 10 KB)

Supplementary file2 (DOCX 94 KB)

Supplementary file3 (DOCX 29 KB)

## Data Availability

All relevant data are within the paper. The full data set will be made available upon request from the corresponding author.

## Footnotes

1 For meta-regression with age as a predictive factor, only 9 studies were included. Gage & Egan (1984) did not report this datum in their article.
