# Supplementary material for: Alexithymia and Hypertension: Does Personality Matter? A Systematic Review and Meta-analysis
Source: Curr Cardiol Rep. 2023 May 22;25(7):711–24. doi: 10.1007/s11886-023-01894-7 (PMC10307708; doi:10.1007/s11886-023-01894-7)
Supplement: Supplementary file 1 — Supplementary file1 (DOCX 10 KB) [file 11886_2023_1894_MOESM1_ESM.docx]

**Articles excluded in the full-text screening**

Anderson C. D. (1981). Expression of affect and physiological response in psychosomatic patients. Journal of psychosomatic research, 25(3), 143–149. <https://doi.org/10.1016/0022-3999(81)90026-x>

Fava, G. A., Baldaro, B., & Osti, R. M. (1980). Towards a self-rating scale for alexithymia: A report on 150 medical patients. Psychotherapy and Psychosomatics, 34(1), 34–39. <https://doi.org/10.1159/000287444>

Greenberg, R. P., & Dattore, P. J. (1983). Do alexithymic traits predict illness? Journal of Nervous and Mental Disease, 171(5), 276–279. <https://doi.org/10.1097/00005053-198305000-00003>

Greenberg, R. P., & O'Neill, R. M. (1988). The construct validity of the MMPI alexithymia scale with psychiatric inpatients. Journal of personality assessment, 52(3), 459–464. <https://doi.org/10.1207/s15327752jpa5203_7>

Greiner, B. A., Krause, N., Ragland, D., & Fisher, J. M. (2004). Occupational stressors and hypertension: a multi-method study using observer-based job analysis and self-reports in urban transit operators. Social science & medicine (1982), 59(5), 1081–1094. <https://doi.org/10.1016/j.socscimed.2003.12.006>

Isaksson, H., Konarski, K., & Theorell, T. (1992). The psychological and social condition of hypertensives resistant to pharmacological treatment. Social science & medicine (1982), 35(7), 869–875. <https://doi.org/10.1016/0277-9536(92)90101-u>

Krystal, J. H., Giller, E. L., Jr, & Cicchetti, D. V. (1986). Assessment of alexithymia in posttraumatic stress disorder and somatic illness: introduction of a reliable measure. Psychosomatic medicine, 48(1-2), 84–94. <https://doi.org/10.1097/00006842-198601000-00007>

Nordby, G., Ekeberg, O., Knardahl, S., & Os, I. (1995). A double-blind study of psychosocial factors in 40-year-old women with essential hypertension. Psychotherapy and psychosomatics, 63(3-4), 142–150. <https://doi.org/10.1159/000288951>

Osti, R. M., Trombini, G., & Magnani, B. (1980). Stress and distress in essential hypertension. Psychotherapy and psychosomatics, 33(4), 193–197. <https://doi.org/10.1159/000287431>

Peters R. M. (2006). The relationship of racism, chronic stress emotions, and blood pressure. Journal of nursing scholarship: an official publication of Sigma Theta Tau International Honor Society of Nursing, 38(3), 234–240. <https://doi.org/10.1111/j.1547-5069.2006.00108.x>

Strauss, E. H. (1988). Specifics of emotionality in psychosomatics. Activitas nervosa superior.
