# Supplementary material for: Alexithymia and Hypertension: Does Personality Matter? A Systematic Review and Meta-analysis
Source: Curr Cardiol Rep. 2023 May 22;25(7):711–24. doi: 10.1007/s11886-023-01894-7 (PMC10307708; doi:10.1007/s11886-023-01894-7)
Supplement: Supplementary file 2 — Supplementary file2 (DOCX 94 KB) [file 11886_2023_1894_MOESM2_ESM.docx]

**a)**


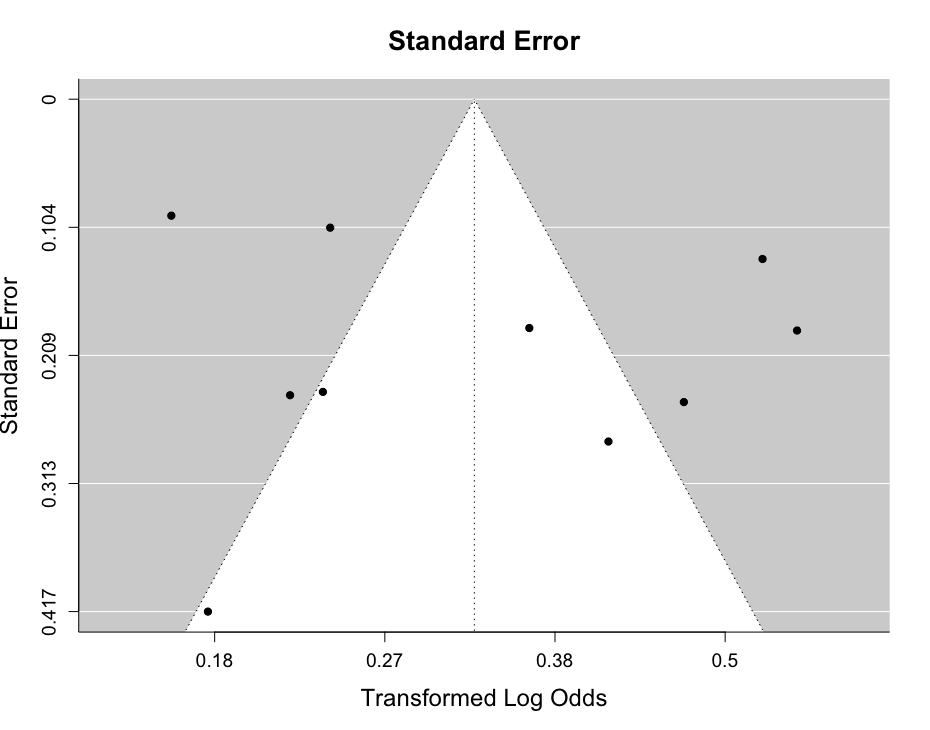


**b)**


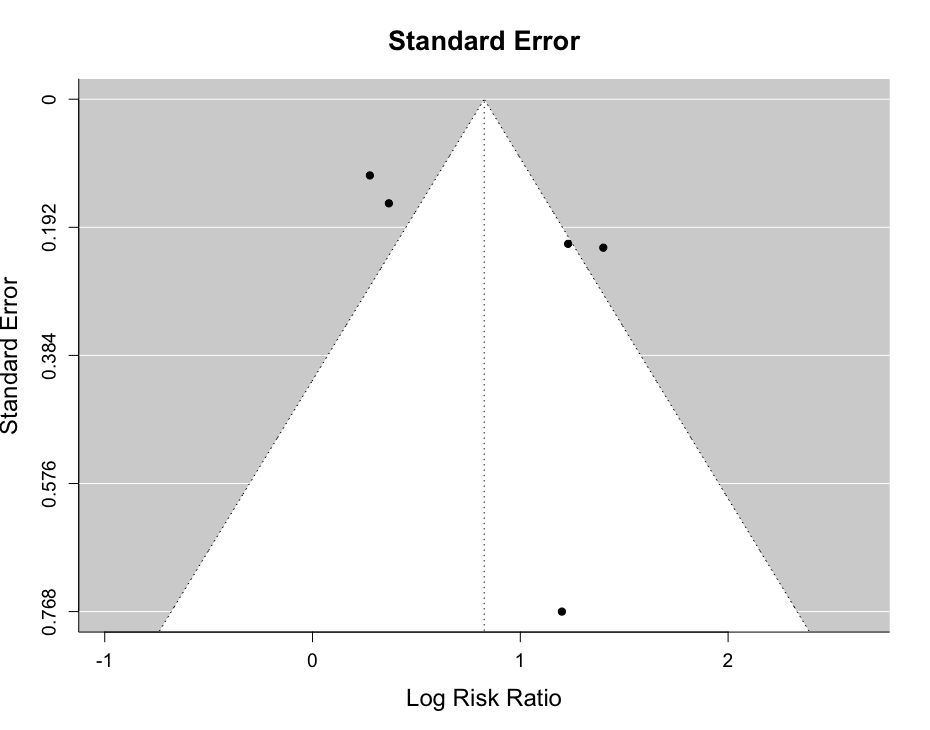


**c)**

**
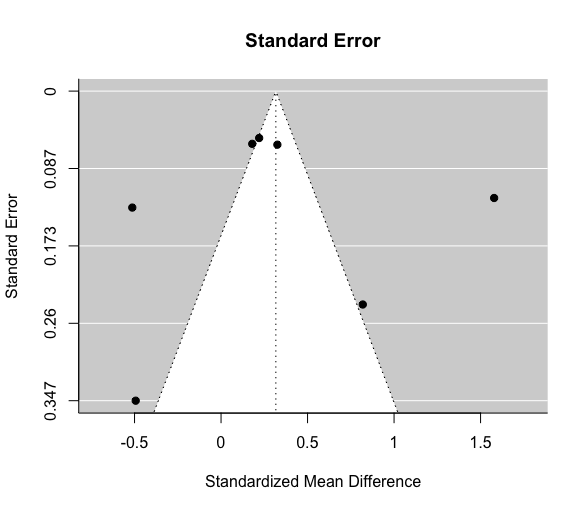
**

**Fig. 1. (a)** Funnel plot for publication bias of alexithymia prevalence among people with HTN. **(b)** Funnel plot for publication bias of alexithymia prevalence among people with HTN vs. without HTN. **(c)** Funnel plot for publication bias of alexithymia men levels among people with HTN vs. without HTN.
