# Supplementary material for: Alexithymia and Hypertension: Does Personality Matter? A Systematic Review and Meta-analysis
Source: Curr Cardiol Rep. 2023 May 22;25(7):711–24. doi: 10.1007/s11886-023-01894-7 (PMC10307708; doi:10.1007/s11886-023-01894-7)
Supplement: Supplementary file 3 — Supplementary file3 (DOCX 29 KB) [file 11886_2023_1894_MOESM3_ESM.docx]

**Quality score based on the JBI assessment checklist for different study designs**

1. **JBI Critical Appraisal Checklist for Studies Reporting Prevalence Data**
2. Was the sample frame appropriate to address the target population?
3. Were study participants sampled in an appropriate way?
4. Was the sample size adequate?
5. Were the study subjects and the setting described in detail?
6. Was the data analysis conducted with sufficient coverage of the identified sample?
7. Were valid methods used for the identification of the   condition?
8. Was the condition measured in a standard, reliable way for all participants?
9. Was there appropriate statistical analysis?
10. Was the response rate adequate, and if not, was the low response rate managed appropriately?

Based on these criteria, the quality score for prevalence study is given below.

| Author (year) | Criteria and corresponding scores | | | | | | | | | Total | % |
| --- | --- | --- | --- | --- | --- | --- | --- | --- | --- | --- | --- |
|  | **#1** | **#2** | **#3** | **#4** | **#5** | **#6** | **#7** | **#8** | **#9** |  |  |
| Gage & Egan (1984) | 1 | 0 | 0 | 0 | 1 | 1 | 1 | 0 | 0 | 4 | 44.44 |
| Paulson et al. (1985) | 1 | 0 | 0 | 1 | 1 | 1 | 1 | 0 | 1 | 6 | 66.67 |
| Rafanelli et al. (2012) | 1 | 1 | 0 | 1 | 1 | 1 | 1 | 1 | 0 | 7 | 77.78 |

1. **JBI Critical Appraisal Checklist for Analytical cross-sectional studies**
2. Were the criteria for inclusion in the sample clearly defined?
3. Were the study subjects and the setting described in detail?
4. Was the exposure measured in a valid and reliable way?
5. Were objective, standard criteria used for measurement of the condition?
6. Were confounding factors identified?
7. Were strategies to deal with confounding factors stated?
8. Were the outcomes measured in a valid and reliable way?
9. Was appropriate statistical analysis used?

Based on the above criteria, the quality score for analytical cross-sectional studies is given below.

| Author (year) | Criteria and corresponding scores | | | | | | | | Total | % |
| --- | --- | --- | --- | --- | --- | --- | --- | --- | --- | --- |
|  | **#1** | **#2** | **#3** | **#4** | **#5** | **#6** | **#7** | **#8** |  |  |
| Casagrande et al. (2019) | 1 | 1 | 1 | 1 | 1 | 1 | 1 | 1 | 8 | 100 |
| Consoli et al. (2010) | 0 | 1 | 1 | 1 | 1 | 1 | 1 | 1 | 7 | 87.50 |
| Grabe et al. (2010) | 1 | 1 | 1 | 1 | 1 | 1 | 1 | 1 | 8 | 100 |
| Hänninen et al. (2011) | 1 | 1 | 1 | 1 | 1 | 1 | 1 | 1 | 8 | 100 |
| Muneta et al. (1997) | 1 | 1 | 1 | 1 | 0 | 0 | 1 | 1 | 6 | 75 |
| Niiranen et al. (2006) | 1 | 1 | 1 | 1 | 1 | 1 | 1 | 1 | 8 | 100 |

1. **JBI Critical Appraisal Checklist for Case controls studies**
2. Were the groups comparable other than the presence of disease in cases or the absence of disease in controls?
3. Were cases and controls matched appropriately?
4. Were the same criteria used for identification of cases and controls?
5. Was exposure measured in a standard, valid and reliable way?
6. Was exposure measured in the same way for cases and controls?
7. Were confounding factors identified?
8. Were strategies to deal with confounding factors stated?
9. Were outcomes assessed in a standard, valid and reliable way for cases and controls?
10. Was the exposure period of interest long enough to be meaningful?
11. Was appropriate statistical analysis used?

The quality measures of case controls studies based on the above listed criteria.

| Author (year) | Criteria and corresponding scores | | | | | | | | | | Total | % |
| --- | --- | --- | --- | --- | --- | --- | --- | --- | --- | --- | --- | --- |
|  | **#1** | **#2** | **#3** | **#4** | **#5** | **#6** | **#7** | **#8** | **#9** | **#10** |  |  |
| Di Trani et al. (2018) | 0 | 1 | 0 | 1 | 1 | 0 | 0 | 1 | 0 | 1 | 5 | 50 |
| Jula et al. (1999) | 1 | 1 | 1 | 1 | 1 | 1 | 1 | 1 | 0 | 1 | 9 | 90 |
| Piotrowska-Półrolnika et al. (2019) | 1 | 1 | 1 | 1 | 1 | 1 | 0 | 1 | 0 | 1 | 8 | 80 |
| Todarelli et al. (1995) | 1 | 1 | 1 | 1 | 1 | 1 | 1 | 1 | 0 | 0 | 8 | 80 |

NB: 1 indicates the article does fulfill the specified criteria

0 indicates the article does not fulfill the stated criteria
